# Pupillomotor Dysfunction and Outcomes After Decompressive Craniectomy in Pediatric Patients

**DOI:** 10.3390/jcm15041459

**Published:** 2026-02-13

**Authors:** Martin Petkov, Aurelia Peraud, Ohad Sharon, Andrej Pala, Christian Rainer Wirtz, Thomas Kapapa, Andreas Pfnür

**Affiliations:** Department of Neurosurgery, University of Ulm, Albert-Einstein-Allee 23, 89081 Ulm, Germany

**Keywords:** decompressive craniectomy, pediatric neurosurgery, herniation, anisocoria

## Abstract

**Background:** Decompressive craniectomy (DC) is a life-saving intervention for refractory intracranial pressure (ICP). While outcomes in adults are well documented, pediatric data, especially concerning pupillomotor dysfunction, remain limited. Anisocoria is generally considered a marker of severe neurological compromise, but its clinical relevance in children undergoing DC has not been adequately studied. **Methods:** We retrospectively reviewed 25 pediatric patients treated with DC between 2004 and 2024. Demographic, radiological and clinical data included age, sex, hospital stay, operative time, etiology, side of craniectomy, preoperative midline (ML) shift, Marshall score, Rotterdam score, Glasgow Coma Scale (GCS) and pupillary status before surgery. Functional outcomes were assessed using the pediatric version of the Glasgow Outcome Scale Extended (pGOS-E) at discharge, after 3 months, 1, 2 and 4 years. **Results:** The majority of patients were school-aged children with a median age of 10 (range 0–17) years. Traumatic brain injury accounted for 16 cases and represented the leading etiology for DC. Pupillomotor dysfunction (anisocoria or bilateral fixed pupillary dilatation) was observed in 15 of 25 patients, 47% of whom died during hospitalization, demonstrating a significant association with in-hospital mortality (*p* = 0.02). However, survivors with primary pupillomotor dysfunction demonstrated a favorable recovery at 12 months with a median pGOS-E of 6 (range 4–8), indicating moderate disability. A preoperative ML-shift > 5 mm was not associated with lower pGOS-E scores during follow-up (*p* > 0.05). Bone flap autolysis was observed in 12 out of 14 children (86%) receiving autologous cranioplasty, and 8 (57%) patients required revision surgery with synthetic material. **Conclusions:** In pediatric patients, pupillomotor dysfunction is associated with higher early mortality but does not reliably exclude favorable long-term outcomes. Compared with adult cohorts, children appear to have a greater potential for neurological recovery, suggesting that severe initial clinical findings alone should not preclude timely surgical intervention.

## 1. Introduction

Decompressive craniectomy (DC) is a neurosurgical procedure for reducing intracranial pressure in patients with traumatic brain injury (TBI), intracranial hemorrhage or extensive cerebral insult [1]. Affected patients often require prolonged intensive care treatment and mechanical ventilation [2]. The decision for surgery depends on factors such as the underlying disease, location of the causative process, comorbidities, preexisting neurological deficits and age of the patient [3]. Although some prognostic factors have been described in the literature, most studies include adult patient collectives [1,3,4]. While the effectiveness of DC has been supported by randomized trials in adults [5,6], data in the pediatric population remain sparse and are largely derived from small case series and retrospective analyses [7]. In children, the causes of increased ICP requiring DC differ somewhat from adults and include a higher proportion of traumatic brain injury compared to adults [8,9].

According to the literature, DC is performed in approximately 16–23% of pediatric patients with severe traumatic brain injury, defined by a GCS of ≤8 [10,11,12,13]. It is considered the ultima ratio for refractory elevated intracranial pressure in children. Neural plasticity in young patients can lead to better functional recovery after traumatic intracranial injury compared to older patient groups [14]. At the same time, the pediatric population is not homogeneous and requires age-specific consideration, as infants and young children constitute distinct subgroups with markedly different ICP dynamics. Neonates and infants under 1 year of age, owing to open cranial sutures and fontanelles, may accommodate increases in intracranial volume with relatively greater compliance, which could delay but not prevent critical rises in ICP [15]. In toddlers between 1 and 4 years, progressive closure of sutures and reduced cranial elasticity coincide with still relatively small subarachnoid and cisternal spaces, limiting compensatory capacity and predisposing to earlier decompensation in the setting of cerebral edema or mass effect. In contrast, school-aged children more closely resemble adults with regard to cranial rigidity, but their reserve capacity remains lower due to proportionally smaller cerebrospinal fluid compartments. These developmental differences imply that a uniform ICP threshold (e.g., 25 mmHg) may not have equivalent consequences for cerebral perfusion across these groups, and certain secondary signs, such as anisocoria in diffuse edema, may not occur in the same way in infants under 6 months [16,17]. Furthermore, ICP values considered normal vary between the different age groups of pediatric patients. Terminal ICP thresholds for 2–6 years were 12 mmHg, for 7–10 years, 18 mmHg, and for 11–15 years, 16 mmHg in a study by Chambers et al. [18]. A critical early sign of neurological deterioration is pupillomotor dysfunction such as anisocoria, which typically signals uncal herniation due to extensive cerebral swelling [16]. Although anisocoria is often associated with poor outcomes in adults, its predictive value in non-adults is less well established.

The prognostic significance of pupillary abnormalities in pediatric patients must be interpreted within the context of age-specific neuroanatomical and physiological conditions, rather than treating “children” as a single homogeneous category, in contrast to adults [10,11].

This study aims to inform clinical decision-making by evaluating the prognostic relevance of pupillomotor dysfunction in pediatric patients undergoing DC, with particular emphasis on whether futility criteria applied in adults, such as denying surgery in the presence of anisocoria or bilateral fixed pupillary dilatation, can be transferred uncritically to children.

## 2. Materials and Methods

A retrospective chart review was conducted at our neurosurgical department, including pediatric patients (age < 18 years) who underwent DC between January 2004 and March 2024. Three types of craniectomies were performed: bifrontal craniectomy, unilateral or bilateral frontotemporoparietal craniectomy. The present cohort also includes patients who underwent primary DC, which was conducted upon clinical requirement immediately after admission and initial cranial imaging, in contrast to secondary DC, which is performed in the further course of therapy. Preoperative pupillary status was assessed as part of the routine neurological examination using a handheld penlight. DC was defined as the removal of a bone flap, followed by duraplasty, to permit outward herniation of swollen brain tissue and thereby reduce intracranial pressure [19]. Inclusion criteria were: (1) age under 18 at the time of surgery, (2) DC performed for increased ICP due to any cause, and (3) availability of clinical and radiological data. Patients with incomplete records, non-decompressive craniectomies and posterior fossa decompressive surgery were excluded.

Elevated intracranial pressure was assessed based on age-adjusted clinical and radiological criteria, including neurological deterioration, midline shift, and ventricular compression. Neurological decline was interpreted according to age-specific physiological responses: in infants and toddlers, by changes in consciousness, irritability, bulging fontanelle, or progressive bradycardia and hypertension; in older children, by a decrease in the Glasgow Coma Scale and the emergence of new focal or pupillary abnormalities. In seven patients, intracranial pressure was additionally quantified using parenchymal probes.

In non-traumatic cases, such as intracranial tumors or ischemic lesions, radiological signs of mass effect were evaluated in conjunction with the clinical course, since structural displacement alone may not necessarily indicate elevated intracranial pressure.

Therapy-refractory intracranial hypertension was defined as persistently increased pressure or progressive neurological decline despite optimized conservative management, including sedation, osmotherapy, and controlled hyperventilation. Data extracted included age, sex, etiology, age-adapted pediatric GCS at admission (used for children 2 years and younger), pupil status before surgery, Marshall classification of traumatic brain injury, Rotterdam score, ML-shift was assessed on axial CT images. The fronto-occipital diameter of the cranial vault was first determined on the slice with the maximal shift. From the midpoint of this diameter, a perpendicular line was drawn to the intracranial midline. The distance between this true midline and the displaced septum pellucidum (or the closest midline structure) was measured in millimeters and defined as the degree of midline shift. Timing and side of DC, ICU and hospital length of stay, mortality, and functional outcome at discharge, three months, one, two and four years using the pediatric Glasgow Outcome Scale Extended (pGOS-E) were assessed. In the analysis we dichotomized the pGOS-E into unfavorable (scores ≤ 4, representing death, vegetative state, or severe disability) and favorable (scores ≥ 5, representing moderate disability to good recovery). This cut-off follows a recent pediatric neuro-outcome study [20].

Statistical analyses included descriptive statistics, Fisher’s exact test for categorical variables, and the Mann–Whitney U test for continuous variables. A multivariable binary logistic regression analysis for a favorable outcome (pGOS-E ≥ 5) was performed. A *p*-value < 0.05 was considered statistically significant. Statistical analyses and graphical illustrations were generated using GraphPad Prism version 10.6.0 (GraphPad Software, San Diego, CA, USA) for macOS. The study was conducted in accordance with the Declaration of Helsinki and received ethical approval from the Ethics Committee of the University of Ulm (reference number 14/25) on 8 April 2025.

## 3. Results

A total of 25 pediatric patients were included in the analysis. The median age was 11 years (range 0–17 years). Age distribution comprised three neonates/infants (0–1 years), three toddlers (2–5 years), eight school-aged children (6–10 years), and 11 adolescents (11–17 years). The most common underlying cause was severe traumatic brain injury (TBI), observed in 16 patients (64%). Additional etiologies included ischemia in four cases (16%), tumors in two cases (8%), spontaneous hemorrhage in one case (4%), and other causes in two patients (8%). Preoperative pupillomotor dysfunction was present in 60% of patients (15/25). Among the six patients presenting with bilateral fixed and dilated pupils, three had traumatic brain injury, two had intracranial tumors, and one had ischemic stroke as the underlying cause. More than half of the cohort exhibited a midline shift greater than 5 mm on initial CT imaging. Eighteen patients underwent unilateral DC, one patient underwent bifrontal DC, and four patients underwent bilateral DC. In three of these cases, bilateral decompression was performed during the initial surgery, whereas one patient required a secondary contralateral decompression due to the development of a subdural hematoma following the initial procedure. Demographic, clinical, and radiological characteristics stratified by etiology are summarized in Table 1. Perioperative complications requiring reoperation occurred in two patients (8%). One patient developed a contralateral subdural hematoma following DC and needed a second DC on the other side and subsequently required wound debridement at a later stage. Another patient underwent surgery for an epidural hematoma at the site of the initial decompression. Four patients required implantation of a ventriculoperitoneal shunt during the course of treatment.

Seven patients (28%) died during their hospital stay. All deaths occurred in patients presenting with pupillomotor dysfunction at admission, corresponding to a mortality rate of 47% (7/15) within this subgroup. Among these, three patients with anisocoria and four patients with bilateral fixed and dilated pupils died.

No deaths were observed among patients with normal pupillary status. Both patient groups, with and without pupillomotor dysfunction, demonstrated improvement during follow-up as assessed by the pGOS-E (Table 2). Remarkably, at the one-year follow-up, the patients who presented with anisocoria or bilateral fixed pupillary dilation achieved a mean pGOS-E of 6, indicating a notably good functional outcome.

**Figure 1:**
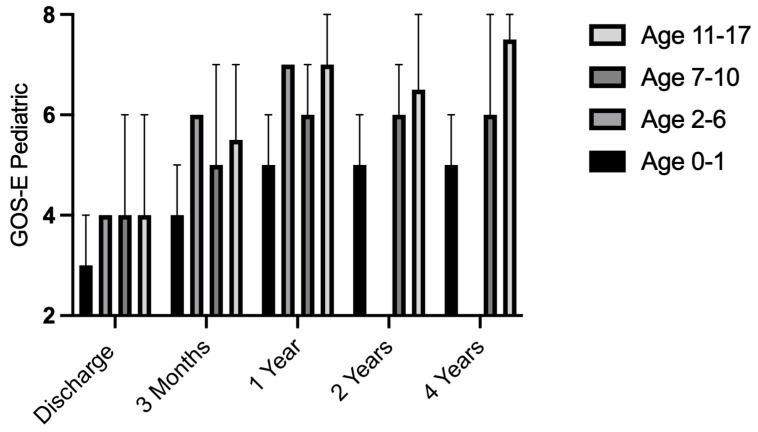
Median pGOS-E values and corresponding ranges across age groups and follow-up intervals. Eighteen patients were evaluated at discharge and 3 months, 16 at 1 year, 12 at 2 years, and 9 at 4 years, with the remaining patients lost to follow-up.

However, at 12-month follow-up, no significant difference in clinical outcome was identified. Even among the six patients who initially presented with bilateral fixed pupillary dilatation, two (33%) survived, and both achieved a favorable outcome after one year, with pGOS-E scores of 6 and 7, respectively. Figure 1 illustrates the clinical course of the entire cohort. Pupillomotor dysfunction was significantly associated with in-hospital mortality in the overall cohort. It did not appear to serve as a reliable predictor of long-term functional outcome among survivors, as favorable recovery remained possible.

Although patients with an initial midline shift greater than 5 mm on CT exhibited higher mortality within our cohort, this difference did not reach statistical significance (*p* = 0.07). At follow-up, no significant difference in clinical outcome was observed between patients with preoperative midline shifts > 5 mm and those with ≤5 mm (Table 3).

Among the 17 patients who underwent cranioplasty, autologous bone flaps were used in 14 cases, whereas the synthetic materials calcium phosphate, polymethylmethacrylate, and polyetheretherketone were used in one case each. Notably, bone flap autolysis occurred in 12 of 14 children (86%) who received autologous cranioplasty, and eight patients required revision surgery with synthetic material due to bone resorption.

Considering the clinical and radiological parameters collected in this study, only the presence of pupillomotor dysfunction was associated with in-hospital mortality. In the multivariable binary logistic regression analysis for favorable functional outcome (pGOS-E ≥ 5), pupillomotor dysfunction showed a trend toward reduced odds of good recovery, although this did not reach statistical significance (OR 0.28, *p* = 0.062). Neither the Glasgow Coma Scale score at admission nor the underlying etiology demonstrated a significant association with outcome (Table 4).

## 4. Discussion

Literature review

DC represents the treatment of last resort for rapid reduction in intracranial pressure in patients with TBI, spontaneous intracranial hemorrhage or ischemic stroke. While several studies have highlighted unilateral or bilateral mydriasis as a negative prognostic indicator in adult patients, the literature concerning pediatric patients remain scarce and is largely limited to small retrospective studies and case series [21,22,23,24]. The aim of the present study is therefore to investigate whether the presence of unilateral or bilateral pupillomotor dysfunction is associated with outcome in pediatric neurosurgical patients and to identify additional factors that may influence the clinical outcome of this vulnerable patient group.

Our findings indicate that anisocoria, even progression to bilaterally fixed pupils, does not uniformly predict a poor outcome in children. Several patients in our cohort with bilaterally dilated, non-reactive pupils survived and achieved neurological recoveries that, while often incomplete, included substantial functional improvement. This is in contrast to the adult trauma literature, where fixed dilated pupils are widely considered an ominous sign of transtentorial herniation and impending brain death [25,26].

Adult series consistently demonstrate that pupillary abnormalities are powerful predictors of poor prognosis after DC. For example, Schröder et al. reported that unilateral fixed pupils increased mortality 1.7-fold in adults, while bilateral fixed pupillary dilatation nearly quadrupled the risk of death. Survival in adults with bilateral fixed pupillary dilatation is rare, with only 8% surviving in that series [27]. Similarly, Tang et al. found that among adult TBI patients with GCS < 5 and bilateral dilated pupils, 79% died within 30 days, and nearly 90% had a poor outcome at six months [28]. As a result, many clinicians consider bilaterally dilated and fixed pupils in adults as devastating status. This perspective is further reinforced by major trials of DC in adults (DECRA, RESCUEicp), which excluded patients with bilateral fixed pupillary dilatation due to their universally poor prognosis [5,6].

In contrast, data for children are limited and there appears to be greater potential for improvement [29]. In our series, one-third of the pediatric patients with bilateral fixed pupillary dilatation survived, a figure far surpassing the adult survival rate.

In a study by Goker et al., one out of three children with preoperatively bilateral fixed pupillary dilatation survived and subsequently underwent cranioplasty during follow-up [30].

The pediatric neurotrauma literature has long alluded to this resilience, as younger patients occasionally recover even after extreme intracranial pressure elevations and herniation. Recent reviews confirm that pediatric patients can achieve better outcomes than adults, likely owing to a combination of greater brain plasticity, fewer comorbidities, and different cerebrovascular responses [12].

Nevertheless, anisocoria in children still indicates a severe injury [30]. In our study, patients with pupillary dilation had lower GCS scores and more severe initial insults than those with symmetric, reactive pupils. This aligns with prior observations that pupillary changes often accompany high intracranial pressure and impending herniation in pediatric TBI. Some series have demonstrated that pupillary status remains a powerful prognostic marker. For example, Jaradat et al. identified pupillary abnormalities as an independent predictor of outcome (*p* = 0.004), with reactive pupils strongly being associated with favorable GOS scores. In their cohort, favorable outcomes (GOS 4–5) were achieved in 67% of early DC, a much higher rate of good recovery than typically reported in adults [31]. Interestingly, the timing of DC, performed within or after 6 h, did not appear to have a significant impact on prognosis, which is consistent with the findings of Nagy et al., where the timing of DC, performed within or after 24 h, was not associated with GOS at discharge or at 3-month follow-up [31,32].

In our series, anisocoria itself did not reach statistical significance as a predictor of long-term outcome, possibly due to our limited sample size or differences in surgical timing and indications. In cohorts with a high proportion of traumatic brain injury, pupillary abnormalities are commonly regarded as indicators of neurological severity or advanced disease state and are closely intertwined with established markers of injury burden, such as low initial Glasgow Coma Scale scores and CT-based grading systems. Accordingly, anisocoria should not be interpreted as an independent causal risk factor for mortality. Rather, it represents an integrated physiological marker of severe primary or secondary brain injury that is associated with higher mortality and therefore functions as a severity- or outcome-related indicator. As such, pupillary status must be interpreted in conjunction with other clinical and radiological findings when informing postoperative prognosis and clinical decision-making.

The survival and functional recovery of children with severe brain injury and signs of herniation emphasize the need for age-specific prognostic indicators and caution against directly extrapolating adult criteria to pediatric practice. Notably, our data contribute to ongoing debates about prognostication in pediatric neurocritical care. Established trauma scores, such as the Pupil Reactivity Score (PRS), may require pediatric-specific recalibration to reflect the greater capacity for recovery of the developing brain [6,33].

Our results also reinforce the concept that maximal therapy, including DC, should be considered even in children presenting with severe clinical deterioration, given their substantial chance of survival and functional recovery. The absence of mortality in our non-anisocoria group further highlights the value of early identification and intervention, while the meaningful recoveries observed among anisocoric survivors argue against therapeutic nihilism in this population.

Bone Flap Autolysis in Pediatric Cranioplasty

**Figure 2:**
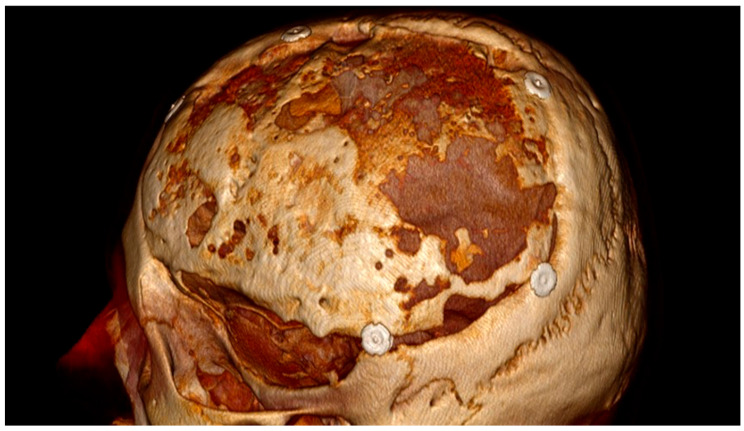
Depicted is a 3D reconstructed CT scan of a male patient, obtained 15 months after autologous bone flap reimplantation, which had been performed 2 months after the initial left hemispheric decompressive craniectomy. The scan demonstrates severe autolytic changes with bone resorption.

As previously described, imaging revealed bone resorption in 12 of 14 patients treated with autologous grafts, of whom 8 required surgical revision. An example of autolysis in our collective is shown in Figure 2. The high rate of autologous bone resorption observed in our cohort mirrors previous reports, which demonstrate children, particularly younger patients, having an increased risk for bone flap autolysis compared to adults [34,35]. Potential risk factors, such as higher rates of bone turnover, ongoing cranial growth, and possible disruption of bone viability following trauma or infection, are discussed in the literature [36,37]. The choice of optimal material for cranioplasty remains a subject of ongoing research, and no consensus has yet been established [38,39,40]. High-quality data in pediatric patients are particularly limited. Pediatric series and systematic reviews report a wide variability in bone flap resorption rates after autologous cranioplasty, ranging from approximately 20% to 80%, with autologous bone being the most frequently used material in children [36,41]. These observations underscore the importance of careful patient selection, diligent follow-up, and further research into biomaterials and strategies to improve bone flap survival in children.

## 5. Limitations

This study has several important limitations. First, its retrospective design carries an inherent risk of selection bias and lacks standardization of surgical indications and perioperative management protocols. Moreover, the absence of a control group, such as pediatric patients managed conservatively for intracranial hypertension, precludes direct comparison and limits the interpretability of the findings.

Assessment of pupillary size and reactivity constitutes an essential component of the neurological examination in patients with suspected intracranial hypertension. In this study, pupillary assessment was based on routine clinical examination and mostly qualitative documentation rather than standardized quantitative pupillometry. Neurological assessments were mostly performed in the emergency department or trauma bay, where quantitative pupillometry was not routinely available. Interobserver variability and subtle pupillary changes may not have been fully captured.

The limited cohort size (25 patients over 20 years) restricts statistical power and increases the potential influence of random variation. As a result, subgroup analyses, including those based on pupillomotor status or radiological thresholds, are underpowered and should be interpreted with caution rather than used to draw definitive conclusions. In addition, the heterogeneity of the cohort must be acknowledged. The inclusion of various etiologies, such as traumatic brain injury, ischemia, tumor, and infection, reflect distinct pathophysiological mechanisms and limits generalizability. The broad age range (0–17 years) introduces variability in neuroplasticity, skull growth, and bone metabolism, which were not stratified in this analysis. Functional outcome assessment relied solely on the pediatric GOS-E because other validated outcome scales were not consistently recorded.

As previously described, the present cohort included both patients undergoing primary decompressive craniectomy, performed immediately after hospital admission, as well as patients treated with secondary decompressive craniectomy during the subsequent course of therapy. However, a systematic and quantifiable assessment of the time interval from admission to surgical decompression was not available and therefore could not be incorporated into the statistical analysis. In adult neurotrauma literature, the timing of DC has been proposed as a relevant factor influencing outcome, reflecting the concept of “time is brain” [6]. Delayed decompression may exacerbate secondary brain injury and has been associated with worse functional outcome in selected adult cohorts, although findings remain heterogeneous and indication-dependent [23,27]. In pediatric patients, however, evidence on the prognostic relevance of surgical timing is limited and inconsistent, with several studies reporting no clear outcome difference between early and delayed decompressive craniectomy [31,32]. Given the present study, the influence of the timing of decompression on outcome could not be reliably assessed.

Furthermore, follow-up in most patients was limited to 12 months and therefore does not account for later neurocognitive development, educational reintegration, or long-term neuropsychological outcomes. Surgical variability represents an additional limitation. Multiple neurosurgeons were involved over the two-decade study period, during which surgical techniques, intraoperative decision-making, and the type of bone flap fixation evolved. These changes, along with differences in the interval to cranioplasty, may have influenced the observed rate of bone flap resorption. The potential influence of ventriculoperitoneal shunt implantation, which occurred in a subset of patients and is known to increase the risk of bone flap resorption, must also be considered. Finally, invasive intracranial pressure monitoring was not performed systematically, and thus, the decision to proceed with DC was not uniformly based on measured ICP values. 

## 6. Conclusions

Anisocoria in pediatric patients undergoing DC is associated with increased early mortality, but not necessarily poor long-term outcome among survivors. These results highlight the unique potential for neurological recovery in children and support a proactive surgical approach in this population, even in cases with severe initial presentations.

## Figures and Tables

**Table 1:** Baseline clinical and radiological characteristics according to underlying etiology. Anisocoria includes unilateral and bilateral fixed pupillary dilatation. ML-shift was measured on axial CT at the level of maximal deviation. Intracranial pressure (ICP) monitor refers to intraparenchymal probes inserted preoperatively. (Abbreviations: N: number of patients; GCS: Glasgow Coma Scale; TBI: traumatic brain injury; IQR: interquartile range; ML: midline; ICH: intracerebral hemorrhage).

Etiology	N	Age Median [IQR]), Years	GCS at Admission Median [IQR]	Anisocoria N (%)	ML-Shift Median [IQR], mm	ICP Monitoring N (%)	In-Hospital Mortality, N (%)
TBI	16	9.5 [6–13.5]	3 [3–4]	8 (50)	4.5 [2–6]	6 (37.5)	2 (12.5)
Ischemic stroke	4	16 [13–17]	3 [3–3]	3 (75)	5.5 [3–7.5]	1 (25)	3 (75)
Tumor	2	5 [1–9]	3.5 [3–4]	2 (100)	12 [11–13]	0 (0)	1 (50)
ICH	1	7 [7–7]	3 [3–3]	0 (0)	11 [11–11]	0 (0)	0 (0)
Other	2	7.5 [2–13]	3 [3–3]	2 (100)	10.5 [8–13]	0 (0)	1 (50)
Total	25	—	—	15 (60)	—	—	7 (28)
	**N**	**Marshall Score, Median [IQR]**			**Rotterdam Score, Median [IQR]**		
TBI only	16	3 [2–4]			3 [2–3]		

**Table 2:** Postoperative outcome at discharge and follow-up period (Abbreviations: N: number of patients; SD: standard deviation; pGOS-E: Pediatric Glasgow Outcome Scale Extended).

Characteristics (Unit)	Proportion/Value
Rate of in-hospital death	28% (7/25)
pGOS-E based on initial pupillary status	
**Discharge**	
Isocoria (N = 10)	
Mean (SD)	3 (1.3)
Median (Range)	2.5 (2–6)
Anisocoria/bilateral fixed pupillary dilatation (N = 15)	
Mean (SD)	3 (2.0)
Median (Range)	2 (1–6)
**3-month follow-up**	
Isocoria (N = 10)	
Mean (SD)	5 (1.4)
Median (Range)	5 (3–7)
Anisocoria/bilateral fixed pupillary dilatation (N = 8)	
Mean (SD)	6 (1.4)
Median (Range)	5.5 (3–7)
**12-month follow-up**	
Isocoria (N = 8)	
Mean (SD)	6 (1.0)
Median (Range)	6 (4–7)
Anisocoria/bilateral fixed pupillary dilatation (N = 8)	
Mean (SD)	6 (1.2)
Median (Range)	6.5 (4–8)

**Table 3:** Mortality and pGOS-E at discharge stratified by midline shift and the presence of pupillomotor dysfunction at admission and follow-up-period (Abbreviations: pGOS-E: Pediatric Glasgow Outcome Scale Extended, ML: midline).

	Pupillomotor Dysfunction	No Pupillomotor Dysfunction	ML-Shift > 5 mm	ML-Shift ≤ 5 mm
Survivors Non-survivors	8/15 (53%) 7/15 (47%)	10/10 (100%) 0/10 (0%)	7/13 (54%) 6/13 (46%)	11/12 (92%) 1/12 (8%)
*p*	0.02		0.07	
Discharge pGOS-E ≤ 4 pGOS-E > 4	11/15 (73%) 4/15 (27%)	9/10 (90%) 1/10 (10%)	11/13 (85%) 2/13 (15%)	9/12 (75%) 3/12 (25%)
*p*	0.6		0.6	
3-month-follow-up pGOS-E ≤ 4 pGOS-E > 4	1/8 (13%) 7/8 (87%)	4/10 (40%) 6/10 (60%)	2/7 (29%) 5/7 (71%)	3/11 (27%) 8/11 (73%)
*p*	0.3		1.0	
1-year-follow-up pGOS-E ≤ 4 pGOS-E > 4	1/8 (12%) 7/8 (88%)	1/8 (12%) 7/8 (88%)	1/6 (17%) 5/6 (83%)	1/10 (10%) 9/10 (90%)
*p*	1.0		1.0	
2-year-follow-up pGOS-E ≤ 4 pGOS-E > 4	0/6 (0%) 6/6 (100%)	1/6 (17%) 5/6 (83%)	0/5 (0%) 5/5 (100%)	1/7 (14%) 6/7 (76%)
*p*	1.0		1.0	
4-year-follow-up pGOS-E ≤ 4 pGOS-E > 4	0/5 (0%) 5/5 (100%)	1/4 (25%) 3/4 (75%)	0/4 (0%) 4/4 (100%)	1/5 (20%) 4/5 (80%)
*p*	0.4		1.0	

**Table 4:** Multivariable binary logistic regression analysis for favorable outcome (pGOS-E ≥ 5) (Abbreviations: GCS: Glasgow Coma Scale, CI: confidence interval).

Variable	Odds Ratio	*p*-Value	95% CI
Pupillomotor dysfunction	0.28	0.062	[0.07; 1.06]
GCS at admission	1.39	0.496	[0.53; 3.67]
Etiology	0.98	0.954	[0.49; 1.95]

## Data Availability

The authors will make the raw data supporting this article’s conclusion available upon request.
